# The Role of Nanomaterials in the Wearable Electrochemical Glucose Biosensors for Diabetes Management

**DOI:** 10.3390/bios15070451

**Published:** 2025-07-14

**Authors:** Tahereh Jamshidnejad-Tosaramandani, Soheila Kashanian, Kobra Omidfar, Helgi B. Schiöth

**Affiliations:** 1 Nanobiotechnology Department, Faculty of Innovative Science and Technology, Razi University, Kermanshah 6714414971, Iran; t.jamshidnejad89@yahoo.com; 2 Department of Surgical Sciences, Division of Functional Pharmacology and Neuroscience, Uppsala University, 75124 Uppsala, Sweden; 3 Biosensor Research Center, Endocrinology and Metabolism Molecular–Cellular Sciences Institute, Tehran University of Medical Sciences, Tehran 1416753955, Iran; omidfar@tums.ac.ir; 4 Sensor and Biosensor Research Center (SBRC), Faculty of Chemistry, Razi University, Kermanshah 6714414971, Iran; 5 Endocrinology and Metabolism Research Center, Endocrinology and Metabolism Research Institute, Tehran University of Medical Sciences, Tehran 1416753955, Iran

**Keywords:** wearable electrochemical biosensors, diabetes mellitus, glucose oxidase, non-enzymatic glucose biosensors, sweat-based biosensors, continuous glucose monitoring, nanomaterials

## Abstract

The increasing prevalence of diabetes mellitus necessitates the development of advanced glucose-monitoring systems that are non-invasive, reliable, and capable of real-time analysis. Wearable electrochemical biosensors have emerged as promising tools for continuous glucose monitoring (CGM), particularly through sweat-based platforms. This review highlights recent advancements in enzymatic and non-enzymatic wearable biosensors, with a specific focus on the pivotal role of nanomaterials in enhancing sensor performance. In enzymatic sensors, nanomaterials serve as high-surface-area supports for glucose oxidase (GOx) immobilization and facilitate direct electron transfer (DET), thereby improving sensitivity, selectivity, and miniaturization. Meanwhile, non-enzymatic sensors leverage metal and metal oxide nanostructures as catalytic sites to mimic enzymatic activity, offering improved stability and durability. Both categories benefit from the integration of carbon-based materials, metal nanoparticles, conductive polymers, and hybrid composites, enabling the development of flexible, skin-compatible biosensing systems with wireless communication capabilities. The review critically evaluates sensor performance parameters, including sensitivity, limit of detection, and linear range. Finally, current limitations and future perspectives are discussed. These include the development of multifunctional sensors, closed-loop therapeutic systems, and strategies for enhancing the stability and cost-efficiency of biosensors for broader clinical adoption.

## 1. Introduction

Wearable sensing technologies have emerged as pivotal tools in enhancing the management of diabetes mellitus by enabling continuous, real-time, and non-invasive monitoring of glucose levels via various skin-accessible biofluids—most notably sweat and interstitial fluid (ISF) [1,2,3,4]. Unlike conventional finger-prick or venous blood sampling methods, these systems offer an upgrade to minimally invasive or non-invasive biosensing for enhancing patient comfort, compliance, and glycemic control [5,6,7]. The integration of bioelectronics, flexible microfluidics, and smart nanomaterials has accelerated the development of compact, user-friendly, miniaturized biosensors with wireless capabilities, enabling personalized feedback loops and improved long-term outcomes [8,9,10,11,12,13,14]. However, normal blood glucose levels in humans typically range from 3.9 to 5.5 mM when fasting, while sweat glucose concentrations are much lower, usually in the micromolar range, with healthy individuals having levels between 60 and 110 μM and diabetic patients having levels between 10 and 1000 μM, which makes glucose monitoring more challenging [15].

Among the latest advances, nanomaterials play a crucial role in increasing the sensitivity, selectivity, and miniaturization of continuous glucose-monitoring (CGM) devices [16]. These nanomaterials, including metal nanoparticles, carbon-based structures, conductive polymers, and nanozymes, serve not only as electrochemical enhancers but also as biocompatible scaffolds that preserve enzymatic activity and enable efficient signal transduction in sweat-based biosensors due to their unique physical, chemical, electrical, optical, and ultimately mechanical properties [17,18,19,20]. Among body fluid such as tears, sweat, saliva, and ISF, sweat is more accessible and suitable for non-invasive sensing due to its easy accessibility, pain-free sampling, and reliable correlation with blood glucose [21]. Thus, it has become a preferred target biofluid in the design of wearable CGM platforms [22].

In response to the growing demand for wearable CGM, Mansour et al. reviewed key elements of wearable biosensors, including sensing mechanisms, energy sources, artificial intelligence (AI) integration, and wireless communication capabilities [16]. They stated that while wireless modules enhance data transfer to external platforms, battery life remains a limiting factor [16]. Furthermore, Kim et al. emphasized the critical role of multifunctional nanomaterials in enabling real-time, selective glucose sensing, and on-demand drug delivery in wearable platforms [23]. Despite these advances, the transition from research to market still faces some obstacles [23]. In this context, Lippi et al. outlined several challenges in the wearable devices, including pre-analytical issues (e.g., cost, patient variability, regulatory barriers), analytical limitations (e.g., calibration, interference, connectivity), and post-analytical concerns, such as misinterpretation of results and inconsistent data integration into clinical records [24].

The present review aims to critically examine recent advancements in nanomaterial-based wearable biosensors for non-invasive CGM, with an emphasis on sweat-based platforms. It explores the roles of nanomaterials in both glucose oxidase (GOx)-based and non-enzymatic sensing platforms, highlights emerging trends, and discusses future pathways for overcoming analytical challenges. Wearable devices’ performance was studied using crucial parameters such as sensitivity, linear range, and limit of detection (LOD), concerning real-life applications.

## 2. Improving Wearable GOx-Based Electrochemical Biosensors Using Nanomaterials

GOx-based electrochemical biosensors have undergone substantial evolution through four distinct generations [25]. The first-generation sensors utilized GOx to oxidize glucose, generating hydrogen peroxide (H_2_O_2_), which was then detected electrochemically; however, they were limited by interference from other electroactive species [25]. Second-generation systems addressed this by incorporating electron mediators such as ferrocene derivatives to shuttle electrons from GOx to the electrode, bypassing the need for oxygen and improve specificity [26]. Third-generation biosensors introduced direct electron transfer (DET) between GOx and the electrode, eliminating the need for mediators and further enhancing response times and selectivity [27].

**Figure 1:**
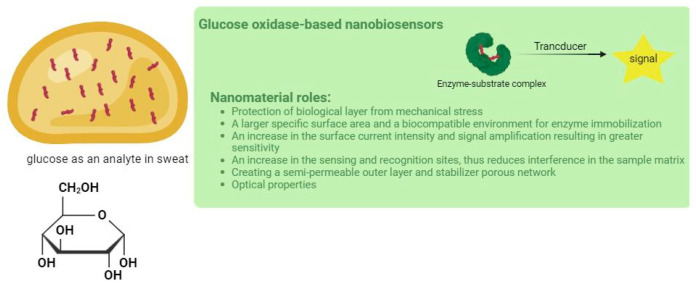
Schematic representation of the roles of nanomaterials in GOx-based wearable electrochemical biosensors for glucose detection.

Currently, the fourth generation represents a transformative leap by integrating advanced functional nanomaterials to create highly sensitive, selective, and miniaturized biosensors suitable for CGM in wearable devices [28]. Despite this progress, sweat encounters challenges due to its less quantity of glucose in comparison to blood, serum, and plasma, as well as the presence of interfering substances like ascorbic acid, uric acid, dopamine, and various electrolytes [29,30,31]. These limitations necessitate more robust and selective sensing strategies. To address these issues, recent advancements have focused on the incorporation of diverse nanomaterials that improve electrochemical properties, enzyme stability, and mechanical resilience of GOx-based biosensors [31,32] (Figure 1).

Nanomaterials can enhance biosensors’ performance primarily by increasing the electrode surface area for enzyme immobilization, enabling protecting the biological recognition layer from degradation and mechanical stress during skin contact [32]. Furthermore, studies show that nanostructured materials, such as gold nanoparticles, metal-organic frameworks (MOFs), graphene derivatives, and conductive polymers, can significantly increase DET, amplifying electrochemical signals, and thus enhance the conductivity and sensitivity of flexible biosensors [33,34,35,36]. This makes them particularly suitable for real-time, non-invasive glucose monitoring in sweat-based wearable systems.

### 2.1. Enhancement of Wearable GOx-Based Electrochemical Biosensors Using Metal Nanomaterials

Metal nanomaterials, such as gold, iron, zinc, and copper, can play a pivotal role in CGM systems. Their nanoscale dimensions result in a high specific surface area, providing abundant active sites that facilitate the adsorption of target molecules. Moreover, their excellent DET capabilities enhance catalytic performance, making them ideal candidates as selective recognition elements for electrochemical signal transduction in glucose sensing applications [29].

In this regard, Dervisevic et al. introduced a wearable glucose-sensing platform utilizing micropillar array (MPA) surfaces, specifically designed to protect the enzyme layer from mechanical stress upon wear [37]. The working electrode was functionalized in a stepwise manner, consisting of an Au-Si-MPA base layer, followed by a Prussian Blue (PB) layer, and a chitosan (Cs)–gold nanoparticle composite layer, onto which GOx was immobilized [37].

Electrochemical measurements revealed a progressive increase in voltammetric peak intensity, along with distinct redox peak shifts, corresponding to each stage of surface modification with PB [37]. The subsequent addition of Cs led to a decrease in peak current, which is consistent with its intrinsic insulating properties [37]. Similarly, the immobilization of GOx further reduced the peak current due to the enzyme’s insulation effect [37]. In contrast, incorporation of highly conductive Au-NPs significantly enhanced the redox peak current [37]. The developed flexible biosensor patch demonstrated a cost-effective, user-friendly, and reliable platform for sweat-based glucose monitoring, offering effective protection of the GOx layer [37]. The sensor exhibited a broad linear detection range, good sensitivity (4.7 ± 0.8 μA mM^–1^), and an appropriate LOD [37] (Table 1).

In addition, a highly flexible sensing platform was developed by integrating a three-dimensional (3D) hierarchical porous Au–hydrogel–GOx electrode with soft micro-electromechanical systems (soft-MEMS) technology [38]. While primarily designed for glucose detection, this system was also tested for lactate monitoring using immobilized lactate oxidase [38]. The resulting universal biosensor exhibited stable mechanical performance, extended operational durability exceeding 15 days, and good selectivity toward both glucose and lactate [38]. Real-time, non-invasive detection of glucose and lactic acid on human skin was successfully demonstrated with the aid of wireless and Bluetooth communication modules [38]. The choice of Au hydrogels was based on their excellent biocompatibility, high catalytic activity, and mechanical flexibility, enabling them to function as both enzyme carriers and active sensing materials [38]. Simultaneously, the use of soft-MEMS technologies contributed to the reproducibility and cost-effectiveness of the Au-based electrodes [38]. The reported sensitivity of this biosensor reached 10.51 μA mM^−1^ cm^−2^ for glucose (Table 1).

Similarly, a miniaturized and flexible biosensor for real-time sweat-based monitoring of glucose and lactate was fabricated using gold nanopine needles (AuNNs) [39]. Enzyme immobilization on the electrode surface was facilitated by poly(ethylene glycol) diglycidylether (PEGDE), a bifunctional cross-linker containing two epoxy groups capable of reacting with the amino groups of enzymes to form a stable immobilization matrix [39]. The deposition of AuNNs significantly increased the surface-to-volume ratio, enhancing enzyme loading capacity [39]. Furthermore, AuNNs served as effective signal amplifiers [39]. Due to its excellent flexibility, the biosensor could be easily bent and attached to human skin for practical wearable applications [39] (Table 1).

Additionally, a study conducted by Zhang et. al. presented a flexible wearable biosensor based on a gallium (Ga)\@MXene hydrogel system for CGM in sweat [40]. The sensor incorporated a 3D conductive and highly stretchable network using liquid metal Ga integrated into MXene, a layered material known for excellent electrical conductivity and flexibility. The MXene hydrogel was combined with CS to improve water absorption and skin adhesion [40]. The resulting biosensor showed a low LOD, high sensitivity, and a wide detection range, making it suitable for real-time glucose monitoring [40]. The porous 3D structure of Ga\@MXene\CS enhanced conductivity and stretchability, enabling the sensor to conform well to skin and maintain stable function during sweat stimulation [40] (Table 1).

Another study conducted by Li et al. presented a flexible, miniaturized glucose sensor using a printable MXene/Fe_3_O_4_ nanocomposite [41]. The nanomaterials played pivotal roles: two-dimensional MXene provided a high surface area, excellent electrical conductivity, and abundant functional groups for robust enzyme immobilization and efficient electron transfer, while aminated Fe_3_O_4_ nanoparticles enhanced enzyme binding through covalent attachment and boosted catalytic activity [41]. Their combination formed a stable, high-viscosity ink suitable for precise dispensing printing [41]. The resulting 5 × 5 mm micro-sensor exhibited a broad detection range, low LOD, and strong anti-interference performance [41]. Validated with real samples, this sensor represented a scalable and cost-effective solution for real-time physiological glucose monitoring [41]. Similarly, Zeng, et al. used a platinum (Pt) single-atom catalyst anchored on nickel oxide (NiO) nanomaterial to enhance electrocatalytic activity and provide a high surface area for efficient enzyme immobilization [42]. This design significantly improved the sensitivity and stability of sweat glucose detection in wearable formats [42] (Table 1).

Lastly, a cost-effective and flexible electrochemical glucose biosensor was developed by immobilizing GOx onto zinc oxide (ZnO) nanoflakes synthesized on a gold-coated stretchable polyethylene terephthalate (PET) film [43]. In this design, the ZnO nanoflakes served to increase the surface charge density, a key factor in facilitating effective GOx immobilization [43]. This configuration enhanced sensing efficiency by eliminating the need for an additional binding layer, thereby enabling a direct and stable interface for rapid electron transfer following enzyme immobilization [43]. The fabricated biosensor demonstrated a sensitivity of 29.97 μA/decade/cm^2^ [43] (Table 1).

### 2.2. Enhancement of Wearable GOx-Based Electrochemical Biosensors Using Carbon Nanomaterials

Different forms of carbon nanomaterials and their hybrids have been extensively investigated for the development of efficient CGM [44]. This is attributed to their inherent properties, such as ease of fabrication, well-established functionalization methods, lightweight nature, suitable thermal stability, excellent mechanical strength, high electrical conductivity, remarkable catalytic activity, and rapid electron transfer kinetics [45]. Furthermore, their large surface area and tunable porosity make them ideal scaffolds for the immobilization of either enzymes or catalytic nanostructures in biosensor fabrication [46,47,48].

Accordingly, Zheng et al. developed a sweat glucose biosensor based on a polyglycolic acid–carbon nanotube (PGA–CNTs) composite film, which offered a high specific surface area for enzyme loading [49]. The porous 3D PGA structure was fabricated using a solvent evaporation technique, followed by CNT deposition to produce the PGA–CNTs electrode, onto which GOx was immobilized through physical adsorption enabled by the expanded surface area of the 3D network [49]. Experimental results indicated that the interconnected mesh-like architecture formed by CNTs on the PGA substrate significantly enhanced charge transfer efficiency and allowed stable entrapment of a large amount of GOx within the electrode matrix [49]. The resulting biosensor exhibited robust conductivity under mechanical stress and demonstrated accurate glucose detection even in the presence of interfering substances [49] (Table 2).

Additionally, to enhance conductivity, catalytic activity, and selectivity, Shamili et al. proposed an all-printed flexible glucose biosensor [50]. The working electrode was screen-printed using a custom-formulated ink containing multi-walled carbon nanotubes (MWCNTs), poly(3,4-ethylenedioxythiophene)\:polystyrene sulfonate (PEDOT\:PSS), and iron (II, III) oxide (Fe_3_O_4_) NPs [50]. The MWCNTs were functionalized with metal oxide nanoparticles and the conductive polymer PEDOT: PSS to enhance the electrochemical performance of the composite, due to their chemical stability, catalytic activity, biocompatibility, ease of preparation, and nontoxic nature [50]. Furthermore, the working electrode was modified with PB-NPs and GOx [50]. The incorporation of PB-NPs enabled faster electron transfer kinetics at low potential and provided high selectivity towards H_2_O_2_ [50]. MWCNT-based screen-printed electrodes (SPEs) demonstrated high sensitivity (~4.495 μA·μM^−1^·cm^−2^), a high rate of electron transfer, and electrocatalytic activity towards glucose oxidation, making them ideal for sweat-based glucose sensing [50]. The results revealed a strong correlation between blood glucose levels and sweat glucose concentrations, indicating the biosensor’s potential for real-time monitoring [50] (Table 2).

Similarly, a sandwich-structured biosensor incorporating PB-NPs and self-assembled MWCNT-COOH on the electrode was reported to enhance the performance of an electrochemical glucose biosensor [51]. GOx was immobilized using Cs as the catalytic unit and encapsulated with Nafion to ensure stable performance [51]. The combination of MWCNT-COOH and PB-NPs contributed to a signal enhancement [51]. The biosensor demonstrated a sensitivity of 11.87 μA·mM^−1^·cm^−2^ (Table 2).

Furthermore, Tong et al. employed Cs and Nafion in a microneedle-based glucose biosensor consisting of carbon black nanoparticles (CB-NPs), PB, and GOx [52]. CB-NPs’ functionalization was due to the excellent electrical conductivity and solvent dispersibility, facile functionalization, numerous defect sites, and fast electron transfer kinetics [52]. In addition, the deposition of PB onto the CB-NPs made the biosensor to have extremely low operating voltages and the Nafion was essential as a cation-exchange polymer membrane that selectively excludes anions such as acids and chlorides from the electrode surface and reduces interference in the sample matrix [52] (Table 2).

Additionally, a three-electrode microneedle electrochemical biosensor and a fully integrated radiochemical analysis system were reported with a long-term performance on diabetic rats [53]. This was achieved by electrodepositing PB and crosslinking GOx and Cs to form a 3D network using glutaraldehyde (GA), resulting in a sensitivity of 8.425 μA·mM^−1^·cm^−2^ [53]. Likewise, Cs role here as the outermost layer of the biosensor was a semi-permeable outer and stabilizing layer, which improved the stability of the whole sensor through its adhesion [53]. Bovine serum protein (BSA) was used to further stabilize and maintain the activity of GOx [53]. The 3D network also facilitates the PB-mediated charge transfer by electrodeposition on graphite-powder-filled working electrode, allowing for fast and highly sensitive analysis of glucose [53] (Table 2).

Moreover, a multi-detection enzymatic biosensor based on an N-doped Graphene Quantum Dots (N-GQDs) anchored polyaniline (PANI) matrix was developed for simultaneous detection of H_2_O_2_ and glucose [54]. The enhanced electron transfer facilitated by N-GQDs enabled the N-GQDs/PANI nanocomposite to offer significantly greater sensitivity for H_2_O_2_ detection than pristine PANI (Table 2) [54]. Likewise, Chiu et al. presented the development of a stable flexible electrochemical biosensor designed for non-invasive glucose monitoring through sweat analysis [55]. A nanocomposite sensor was fabricated by integrating carbon nitride quantum dots (CNQDs) with polyaniline (PANI), resulting in improved electrochemical performance [55]. The CNQDs, known for their high surface-to-volume ratio and abundant edge sites, contributed to enhanced sensitivity in PANI by facilitating effective charge transfer and ion transport [55]. Moreover, the presence of pyridinic nitrogen groups within the CNQDs improved PANI’s conductivity under neutral pH by enabling proton retention and generating surface negativity [55]. The developed sensor exhibited excellent sensitivity and mechanical durability, maintaining its performance even after repeated bending cycles without any noticeable structural damage in the composite layer [55] (Table 2).

Another flexible biosensor, termed GOx/Cs/GS/PB (GCGP), was developed utilizing glucose oxidase (GOx), chitosan (Cs), a graphene sponge (GS), and Prussian Blue (PB) for the purpose of sweat glucose monitoring [56]. The GS component, with its high specific surface area and interconnected porous architecture, facilitated efficient absorption and uniform distribution of substantial amounts of GOx [56]. In addition, the excellent electrical conductivity of GS allowed for the formation of multiple conductive pathways, supporting rapid electron and ion transfer [56]. Its rich availability of binding sites also made GS a suitable scaffold for integration with other functional materials [56]. This biosensor demonstrated a sensitivity of 1790 nA·mM^−1^·cm^−2^, indicating strong potential for non-invasive glucose detection [56] (Table 2).

Additionally, Moradi et al. introduced a paper-based origami glucose biosensor, in which graphite pencil electrodes were directly sketched onto the paper substrate [57]. To enhance the paper’s electrical conductivity and facilitate electron transfer from the GOx to the electrodes, a mediator consisting of MWCNTs and Ti_3_C_2_, dispersed in PEDOT:PSS ink, was applied to the mediator region. This mediator ink was synthesized through a straightforward, single-step procedure, eliminating the need for advanced instrumentation or elaborate synthesis methods [57]. Furthermore, the composite mediator effectively lowered the required operating potential for glucose oxidation, attributed to its high surface area, distinctive electronic characteristics, and strong catalytic performance [57].

### 2.3. Enhancement of Wearable GOx-Based Electrochemical Biosensors Using Other Nanomaterials

Conductive hydrogels are soft materials resemble semiconductors with a porous 3D network structures [58]. They are gaining attention for future wearable devices design owing to their cost-effectiveness, excellent flexible microstructural configuration properties, biomimetic characteristics, extensive specific surface area, high stability, electrical conductivity, self-healing capabilities, and unique biocompatibility [58,59]. In this context, Pan et al. introduced an innovative electrochemical biosensor for non-invasive CGM, utilizing a conductive hydrogel composed of PEDOT:PSS functionalized with MXene. During fabrication, ethylene glycol (EG) was incorporated into the hydrogel to promote polymer chain extension, which resulted in enhanced electrical conductivity, improved porosity, and superior film-forming capability, while also increasing the material’s flexibility and structural stability by improving the stacking and peeling problems commonly observed in powdered materials [60]. The inclusion of MXene significantly enhanced the conductivity of the PEDOT:PSS hydrogel due to its high electron transfer efficiency. The hydrogel solution, containing GOx, was used to modify the electrode surface upon solvent evaporation, which formed glucose-responsive films [60]. The 3D honeycomb-like architecture of the resulting hydrogel, combined with the presence of MXene, created abundant electron transfer pathways and catalytic sites [60]. The biosensor demonstrated reliable glucose detection in agreement with standard glucose meters, achieving a sensitivity of 21.7 μA·mM^−1^·cm^−2^ [60] (Table 3).

Furthermore, the integration of physical activity monitoring with sweat glucose measurement opens the door to next-generation multifunctional non-invasive health-monitoring systems [61]. In this regard, a dual-network hydrogel sensor was developed using sodium carboxymethyl cellulose to fabricate a high-performance nanocomposite hydrogel with excellent flexibility [61]. This biosensor enabled simultaneous tracking of body motion and sweat glucose levels [61]. Incorporation of GOx-thioglycolic acid-gold nanoparticle (GTAN) hybrids into the hydrogel provided remarkable stability and electrocatalytic performance for glucose sensing in sweat [61]. The sensor achieved a sensitivity of 0.02571 μA·μM^−1^·cm^−2^ [61] (Table 3).

Last but not least, Komkova et al. reported a wearable glucose biosensor fabricated by drop-casting, based on the bioelectrocatalytic activity of pyrroloquinoline quinone-dependent glucose dehydrogenase (PQQ-GDH) [62]. To ensure proper orientation of the immobilized enzyme, poly(methylene green) (p(MG)) nanoparticles were employed as anchoring agents [62]. The obtained ratio was 2.5 times lower than that for biosensors based on electropolymerized p(MG) films and practically an order of magnitude lower than that for the best reagentless sensors based on PQQ GDH immobilized over conductive nanomaterials [62]. Despite this, the high-efficiency bioelectrocatalysis observed allowed the sensor to function with notable sensitivity even at 0.0 V, making it suitable for both bioelectric power generation and real-time monitoring of sweat glucose variations in non-invasive diabetes management strategies [62] (Table 3).

## 3. The Application of Nanomaterials in Wearable Non-Enzymatic Electrochemical Diabetes Biosensors

In the fourth generation of glucose biosensors, the enzymatic component can be either supplemented or replaced with advanced nanomaterials and technologies to enhance the performance of biosensors, particularly in terms of the stability of the biological layer [63]. These biosensors, which often adopt hybrid strategies, retain the enzymatic specificity for glucose detection while benefiting from the integration of nanomaterials to improve performance [64]. By facilitating DET between the enzyme and the electrode, nanomaterials eliminate the need for mediators, thereby enhancing both sensitivity and response time [65]. The primary innovation in this generation lies in improving the function of enzymatic biosensors through nanotechnology and advanced materials, rather than fully eliminating the enzyme [66]. This approach warranty the proven specificity and reliability of enzymatic systems [66].

**Figure 2:**
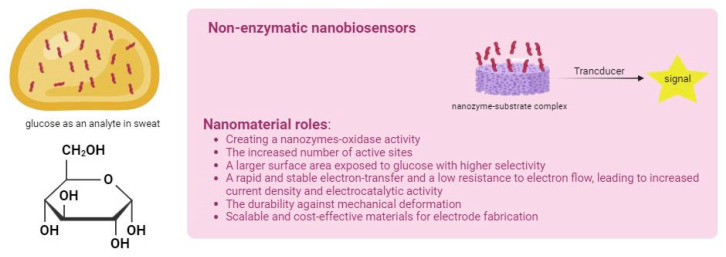
Schematic representation of the roles of nanomaterials in non-enzymatic wearable electrochemical biosensors for glucose detection.

However, the electrochemical oxidation of glucose involves complex processes, including adsorption, electron transfer, and subsequent chemical rearrangements, all occurring on the electrode surface [67]. Nanomaterials can mimic the redox microenvironment of native enzyme systems, allowing glucose to more effectively interact on the surface of biosensor [68]. Moreover, introducing appropriate surface functional groups on NPs facilitates strong interactions between the biomolecules and the NPs [69]. Due to their excellent conductivity, NPs serve both as signal-generating probes and signal enhancers [70]. Non-enzymatic glucose biosensors rely on metal or metal oxide nanomaterials for which atoms act as electrocatalysts in the glucose oxidation reaction, essentially mimicking the catalytic role of natural enzymes [71] (Figure 2). Recent research has increasingly focused on the development of such non-enzymatic biosensors by employing a wide range of nanomaterials and nanocomposites to modify the electrode surface [72].

Currently, there is significant ongoing research into non-enzymatic glucose biosensors that utilize nanomaterials to detect glucose via catalytic mechanisms, independent of GOx [73]. These sensors are designed to overcome limitations related to enzyme instability and degradation, potentially extending the operational lifespan of the biosensors [74]. However, despite advancements in non-enzymatic fourth-generation glucose biosensors, several challenges remain to be addressed in future studies. The key issues include achieving sufficient sensitivity, selectivity, and biocompatibility of the nanomaterials used [75]. Overcoming these challenges is critical for enabling the widespread practical application of fourth-generation wearable non-enzymatic electrochemical diabetes biosensors.

To address these challenges, recently, metal, metal oxidase, and advanced composite nanomaterials have attracted increasing attention in non-enzymatic glucose-sensing platforms [67]. Relatedly, Li et al. introduced a non-enzymatic wearable biosensor designed for real-time glucose monitoring in human sweat [76]. The sensor utilized a hydrogel matrix of poly(2-hydroxyethyl methacrylate) (PHEMA) modified with copper nanoparticles (CuNPs), which served as electrocatalytic sites for glucose oxidation [76]. This design eliminated the need for enzymatic components, enhancing the sensor’s stability and reducing potential degradation over time [76]. The biosensor exhibited a wide linear detection range, with a low LOD, making it suitable for detecting physiological glucose levels in sweat [76]. Additionally, the flexible and stretchable nature of the PHEMA hydrogel ensured skin conformity, making the sensor promising for CGM in wearable health devices [76].

A notable example of non-enzymatic glucose sensing involves the application of advanced composite structures incorporating nanoporous CuO, CuO/Ag, and CuO/Ag/NiO [77]. In this work, the surface of bare glassy carbon electrodes (b-GCEs) was modified with dispersions of CuO–GCE, CuO/Ag–GCE, and CuO/Ag/NiO–GCE to construct the sensing interfaces [77]. Upon immersion in a NaOH solution, the CuO layer functioned as the catalytic component, facilitating glucose oxidation [77]. The silver layer enhanced charge transfer between CuO and NiO, improving electron transfer, while the NiO layer served as the active site for glucose oxidation [77]. Among the tested configurations, the CuO/Ag/NiO-based electrode demonstrated outstanding sensing capabilities, achieving an ultrahigh sensitivity of 2895.3 μA·mM^−1^·cm^−2^ [77] (see Table 4). These findings highlight the great potential of nanoporous CuO/Ag/NiO composites for early, non-enzymatic detection of hyperglycemia [77].

Furthermore, Daboss et al. introduced a flow-through multi-biosensor capable of detecting both glucose and lactate simultaneously, employing Prussian Blue nanoparticles (PB-NPs) as the key transducing element [78]. They developed a core–shell nanozyme structure combining PB with nickel hexacyanoferrate, which resulted in a highly stable and responsive hydrogen peroxide transducer with a sensitivity of 0.20 ± 0.01 A·M^−1^·cm^−2^ [78]. This stabilized configuration contributed significantly to the overall durability of the biosensor system [78]. In a related study, Quan-Fu et al. presented a flexible, wearable, non-enzymatic electrochemical sensor for CGM in sweat, utilizing a hybrid catalyst composed of Pt NPs supported on MXene (Pt/MXene) nanosheets [79]. They further enhanced the sensor’s stability by embedding the Pt/MXene hybrid into a conductive hydrogel matrix, optimizing both mechanical flexibility and signal reliability [79] (Table 4).

Pavadai et al. employed nickel-based MOFs, specifically 3D nickel trimesic acid frameworks (3D Ni-TMAF), which were assembled layer-by-layer on a highly porous nickel substrate, to construct a robust enzyme-mimetic electrochemical biosensor for glucose detection [80]. This system provided a stable, cost-effective, and high-performance alternative to conventional enzyme-based biosensors [80]. The 3D Ni-TMAF structure featured crystalline nanospheres with high porosity and abundant catalytic sites, while the nickel centers facilitated DET, resulting in high sensitivity and rapid response times [80].

Moreover, to address the growing demand for non-invasive CGM, Luo et al. developed a patch-type electrochemical glucose sensor designed for sweat analysis, based on electrospun polyurethane (PU) fibrous mats [81]. The fabrication process involved electrospinning, followed by gold deposition via magnetron sputtering to enhance conductivity and in situ ultrasonic-assisted electrodeposition of Pt nano–pine needle structures along the fibers [81]. The unique combination of PU’s elasticity, the fibrous porous morphology, and the high electrochemical surface area yielded a sensor with notable advantages, i.e., mechanical stretchability (stable up to 10% strain), high sensitivity (203.13 μA·mM^−1^·cm^−2^), low detection limit, high selectivity, and excellent breathability for wearable applications [81] (Table 4).

**Figure 3:**
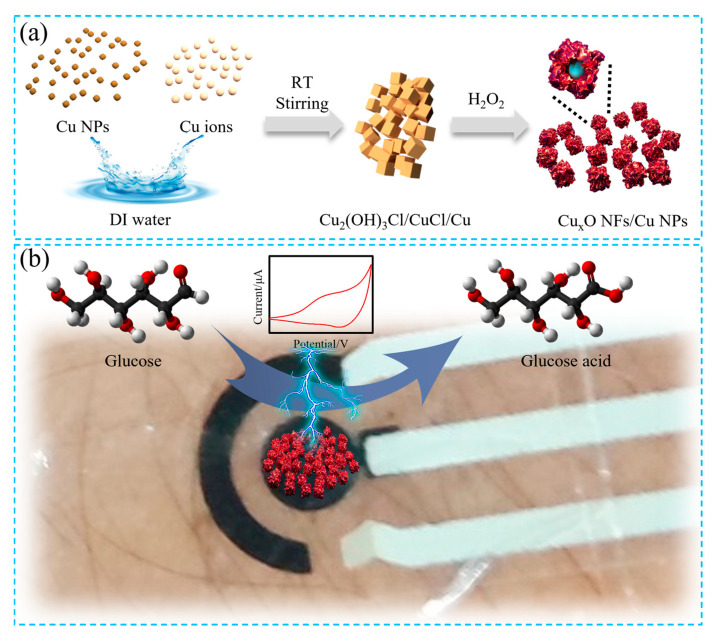
Schematic illustration of the synthesis process of CuxO NFs/Cu NPs nanocomposites (**a**) and their application mechanism in non-invasive glucose detection (**b**), Figure reproduced from [82] Used under the terms of the Creative Commons Attribution (CC BY) license.

Copper oxides (Cu_x_O) indicate outstanding electrochemical activity and thus have a sufficient capacity for non-enzymatic glucose oxidation [72]. Accordingly, Yu et al. developed Cu_x_O nanoflakes (NFs)/Cu NPs nanocomposites to serve as the sensing materials for non-invasive wearable glucose biosensors with a high surface area [82]. They involved CuCl_2_ to enhance the oxidation of Cu NPs to generate Cu_2_O/CuO NFs on the surface to help generating abundant active sites [82]. Due to more active sites, the as-prepared sample exhibited high sensitivity for wearable sweat sensing [82]. Interestingly, the sensitivity of Cu_x_O NFs/Cu NPs-based sensor was three-fold higher than that of CuO NFs/Cu NPs nanocomposites synthesized without CuCl_2_ [82]. Combined with high selectivity and the durability of mechanical deformation, the CuxO NFs/Cu NPs-based biosensor could detect the glucose level change of sweat effectively [82] (Figure 3).

Another study by Su et al. introduced a non-enzymatic, wearable glucose biosensor that integrated CuO nanoparticles with laser-induced graphene (LIG) to enable CGM in sweat [83]. Instead of enzymatic part, CuO serves as the catalytic nanomaterial, facilitating direct redox reactions with glucose in alkaline sweat environments [83]. The biosensor exhibited a fast response time of 600 ms, and excellent stability and selectivity, making it ideal for low-cost, on-body health-monitoring applications [83]. Furthermore, Kim et al. developed an innovative flexible glucose sensor constructed from a composite of copper nanoparticles (CuNPs), laser-induced graphene fibers (LIGF), and a porous LIG network, all integrated onto a polyimide film [84]. This CuNP/LIGF/LIG-based sensor demonstrated notable performance, including high sensitivity, a low LOD, a broad linear response range, and stable functionality under various bending conditions [84].

On the other hand, Zhou et al. developed highly sensitive, non-enzymatic electrochemical glucose biosensors utilizing hollow Fe_3_O_4_ nanospheres (Fe_3_O_4_NSs) immobilized on flexible fiber substrates [85]. These hollow nanospheres, characterized by their unique morphology and excellent catalytic activity, provided an effective solid–liquid interface for efficient mass transport and offered numerous active sites to promote glucose oxidation fibers [85]. Additionally, the formation of rounder and rougher Fe_3_O_4_NSs with near-Gaussian surface characteristics achieved by increasing the Fe^3+^ ion concentration was found to enhance sensor performance [85]. The optimized sensor achieved a sensitivity of 96.1 ± 5.4 μA·mM^−1^·cm^−2^ across a 0–18.0 mM range, with a low detection limit of 19.2 μM [85].

Beside metal and metal oxidase nanoparticles, Sobahi et al. introduced a non-enzymatic electrochemical glucose biosensor based on polyaniline nanofibers [86] (Table 4). The polyaniline-modified electrode demonstrated excellent glucose-sensing capabilities, delivering high sensitivity while maintaining selectivity in the presence of interfering substances [86]. The same study showed it is also cost-effective and does not require complex sample preparation steps [86].

Despite their advantages, such as enhanced stability, cost-effectiveness, and simplified fabrication, non-enzymatic CGM devices based on metallic NPs face important limitations that must be addressed for wearable applications. First, concerns regarding metallic NPs toxicity have provoked the adoption of biocompatible coatings, encapsulation techniques, and safer materials like gold or Pt alloys to reduce leaching and cytotoxic effects particularly in implantable or prolonged skin-contact platforms [87]. Second, sensor lifetime is inherently longer compared to enzymatic counterparts; however, longevity is still constrained by oxidation of active sites and surface fouling [88,89]. Numerous reported devices maintain functionality for several days to weeks under physiological conditions [90]. Advances in nanoporous architectures, bimetallic alloys, and surface modification strategies have improved resilience against fouling and maintained selectivity in complex biofluids [91,92,93]. Recent advances have focused on structural modifications to enhance their functionality in wearable applications with practically biocompatible designs (Table 4).

## 4. Future Perspective in Nanomaterial Applications in Wearable Biosensor for Diabetes Detection

Recent advancements in nanotechnology have significantly contributed to the evolution of wearable CGM devices [4]. Nanomaterials offer remarkable sensitivity and accuracy owing to their unique physicochemical properties, including high surface area and tunable porosity [94]. Current trends focus on the integration of high-performance nanomaterials, such as carbon-based nanostructures, metal nanoparticles, polymeric matrices, and advanced nanocomposites, into flexible, breathable, and biocompatible platforms capable of non-invasive CGM, without the associated risks of infection or user discomfort [95,96,97]. These materials enhance the electrochemical performance of miniaturized wearable devices, enabling real-time, contact-based detection and seamless data transmission to remote or on-site smart systems [98].

Future directions in this field include the development of multifunctional biosensors capable of simultaneous monitoring of multiple biomarkers, improved biocompatibility, prolonged operational lifespan, and the creation of integrated closed-loop systems capable of both sensing and therapeutic delivery [99]. Moreover, ongoing advancements in nanotechnology, alongside interdisciplinary integration, are expected to reduce production costs and improve the accessibility of these advanced wearable biosensing platforms to a wider population [100].

## 5. Discussion

The integration of nanomaterials into wearable glucose biosensing platforms has significantly advanced the performance and applicability of both enzymatic and non-enzymatic electrochemical biosensors. In enzymatic systems, nanomaterials, such as gold nanoparticles, carbon nanotubes, and conductive hydrogels, provide a high surface area for enzyme immobilization and to enhance DET and improve the mechanical compliance of biosensors, all of which are critical for wearable biosensors. These enhancements contribute to improved sensitivity, selectivity, and signal stability in sweat-based CGM. Still, enzymatic biosensors remain susceptible to biological degradation, limited shelf life, and sensitivity to environmental variations, such as temperature, pH, and biofluid composition.

On the other hand, non-enzymatic glucose biosensors circumvent enzyme-related instability by utilizing metal and metal oxide nanostructures (e.g., CuO, NiO, Pt, Fe_3_O_4_) as electrocatalysts for glucose oxidation. These platforms offer enhanced durability, longer operational life, and simplified fabrication procedures. However, achieving high biocompatibility in non-enzymatic sensors continues to be challenging.

An emerging solution is the development of hybrid sensors that combine enzymatic specificity with nanocatalyst-enhanced signal transduction, offering a balance between biological accuracy and operational robustness. In addition, designs such as microneedle arrays, origami paper sensors, and soft hydrogels have contributed to the advancement of wearable biosensors that are flexible, breathable, and minimally invasive. Despite these promising developments, several barriers must be addressed before widespread clinical implementation. These include signal interference from co-existing biomolecules in sweat, sensor stability and durability over prolonged use, scalability, and cost-effectiveness.

A critical limitation in the comparative evaluation of wearable glucose biosensors lies in the non-standardized reporting of analytical parameters, such as selectivity, recovery, and reproducibility. These metrics are frequently derived using heterogeneous methodologies, variable sample sizes, and inconsistent testing protocols, thereby impeding direct and meaningful comparisons of biosensor performance across different studies. Future research needs to focus on interdisciplinary collaboration among materials scientist and biomedical engineers to improve the selectivity and reproducibility of non-enzymatic sensors and to extend the functional lifespan of enzymatic platforms, as well as the integration of real-time analytics and wireless communication. Additionally, designing multifunctional biosensors capable of simultaneously tracking multiple biomarkers, and realizing closed-loop systems that couple sensing with therapeutic delivery, will be crucial to translating laboratory-scale innovations into affordable, user-friendly, and clinically validated devices. This will further enable personalized diabetes management and mark a significant leap toward next-generation diabetes management.

## Figures and Tables

**Table 1:** Diabetes wearable glucose oxidase (GOx)-based electrochemical biosensors performance incorporating metal nanomaterial.

Detected Analytes	Recognition Unit	Linear Concentration Range (mM)	LOD ^1^ (μM)	Sensitivity (μA mM^−1^ cm^−2^)	Reproducibility (%)	Recovery (%)	Nanomaterials Application	Ref.
glucose	Au-Si-MPA ^2^, PB ^3^, Cs-Au NPs ^4^, Gox ^5^	0.05–1.4	26 ± 5	4.7 ± 0.8 μA mM^−1 †^	∼5.3 (n = 10)	NR ^6^	protection of biological layer from mechanical stress, increased surface area, an increase in current intensity	[37]
glucose and lactate	porous Au-hydrogel/GOx and lactate oxidase	0–5.0 for glucose	17.84 for glucose and 11,600 for lactate	10.51	0.30–0.70 (n = 9)	NR	high-mechanical performance and larger specific surface area	[38]
glucose and lactate	AuNNs ^7^, PEGDE ^8^, GOx and lactate oxidase	0–0.25 for glucose and 0–25 for lactate	7 for glucose, 54 for lactate	NR	2.5 for glucose and 4.1 for lactate (n = 5)	92.8–108 for glucose and 98.7–106% for lactate	signal amplification and larger surface area for enzyme immobilization	[39]
glucose	Ga ^9^\@MXene hydrogel, GOx	0.001–1	0.77	1.122 × 10^9^	0.16 (n = 5)	95–107.1	create a conductive and stretchable network	[40]
glucose	MXene/Fe_3_O_4_ nanocomposite, GOx	0.1–10	45.84	Low conc. range: 11.07 × 10^9^ High conc. range: 2.42 × 10^9^	NR	NR	structural support for biocompatible enzyme immobilization, signal transduction	[41]
glucose	Pt single-atom catalyst anchored on NiO nanomaterial, GOx	0–2	3.74	0.003744 µA ^†^	3.03 (n = 5)	NR	To enhance electrocatalytic activity and provide a high surface area for efficient enzyme immobilization	[42]
glucose	ZnO ^10^ nanoflakes, synthesized on an Au-coated PET ^11^ film/GOx	0.1–10	up to 1	29.97μA/decade/cm^2 †^	NR	NR	elevated surface charge density and an effective surface for the immobilization of GOx	[43]

^1^ LOD: limit of detection, ^2^ MPA: micropillar array ^3^ PB: Prussian Blue, ^4^ NPs: nanoparticles, ^5^ GOx: Glucose oxidase, ^6^ NR: not reported, ^7^ AuNNs: gold nanopine needles, ^8^ PEGDE: poly (ethylene glycol) diglycidylether, ^9^ Ga: gallium, ^10^ ZnO: Zink Oxide, ^11^ PET: polyethylene terephthalate. † Reported values of sensitivity are based on different unites (for example logarithmic (decade) concentration changes or just I (μA)), not directly comparable to linear-scale sensitivities (μA mM^−1^ cm^−2^).

**Table 2:** Diabetes wearable glucose oxidase (GOx)-based electrochemical biosensors performance incorporating carbon-based nanomaterials.

Detected Analytes	Recognition Unit	Linear Concentration Range (mM)	LOD ^1^ (μM)	Sensitivity (μA mM^−1^ cm^−2^)	Reproducibility (%)	Recovery (%)	Nanomaterials Application	Ref.
glucose	PGA-CNTs ^2^/Gox ^3^	0.002–0.3	5.18	78.45	1.46 (n = 5)	103.83–109.54	increased the surface area and uniformly immobilized the GOx	[49]
glucose	MWCNT ^4^, PEDOPT: PSS ^5^ hydrogel and iron (II, III) oxide NPs ^6^/GOx	0.001–0.4	~0.38	~4495	2.76 (n = 5)	96.0–98.6	increased the surface area and provides a biocompatible environment for sensitive glucose detection based on GOx	[50]
glucose	GOx/PBNPs ^7^/MWCNT-COOH/GOx	0.01–1	7	11.87	5.81 (n = 5)	94.55–109.92	stabilized GOx and improved electrochemical performance	[51]
glucose	CBNPs-PB ^8^/GOx	0.005–1.25	4.83	14.64	2.1 (n = 3)	101.62–107.94	increase the conductivity and reduces interference in the sample matrix	[52]
glucose	PB, Cs ^9^, GA ^10^ & GOx	0–36	6.44	8.425	3.62 (n = 7)	NR ^11^	creating a semi-permeable outer layer and Stabilizer	[53]
H_2_O_2_ and glucose	N-GQDs ^12^ anchored PANI ^13^ matrix and GOx	0–1 for H_2_O_2_ and 0–0.5 for glucose	34 for glucose	44.06 ± 2.1	Negligible (n = 5)	95.7	the enhanced electron transfer resulting in greater sensitivity	[54]
glucose	CNQDs/PANI ^14^ nanocomposite/GOx	0–0.5	0.029	49.71 ± 0.45	NR	96.27	high surface area and edge-rich architecture, enhanced electron transfer, high mechanical stability	[55]
glucose	GOx/Cs/GS/PB	0.00817–1	2.45	1790 nA·mM^−1^·cm^−2^	NR	NR	the large surface area and the cross-linked hierarchical porous structure of GS enable easy absorption and even distribution of a large amount of GOx	[56]
glucose	MWCNTs/Ti_3_C_2_, dissolved in PEDOT:PSS ink/GOx	0.01–0.4	7	NR	0.3–0.76 (n = 4)	94.6–98.68	enhanced electron transfer, higher surface aera	[57]

^1^ LOD: limit of detection, ^2^ PGA-CNT: polyglycolic acid-carbon nanotubes, ^3^ GOx: Glucose oxidase, ^4^ MWCNT: multi-walled carbon nanotube, ^5^ PEDOPT: PSS: poly (3,4-ethylenedioxythiophene) polystyrene sulfonate, ^6^ NPs: nanoparticles, ^7^ PBNPs: Prussian blue nanoparticles, ^8^ CBNPs-PB: carbon black Nanoparticles-Prussian blue, ^9^ Cs: Chitosan, ^10^ GA: Glutaraldehyde, ^11^ NR: not reported, ^12^ N-GQDs: N-doped Graphene, ^13^ PANI: polyaniline, ^14^ GQD: Graphene Quantum Dots.

**Table 3:** Diabetes wearable glucose oxidase (GOx)-based electrochemical biosensors performance incorporating other nanomaterials.

Detected Analytes	Recognition Unit	Linear Concentration Range (mM)	LOD ^1^ (μM)	Sensitivity (μA⋅mM^−1^⋅cm^−2^)	Reproducibility (%)	Recovery (%)	Nanomaterials Application	Ref.
glucose	MXene-functionalized PEDOT:PSS ^2^ conductive polymer hydrogels/Gox ^3^	0.003–0.094	1.9	21.7	NR ^4^	NR	Creating porous network for GOx, higher conductivity and stability (91.19% electrode response after 10 days)	[60]
glucose	CMC-Na-GTAN ^5^/GOx	0–120	0.28	25.71	NR	92.6	Creating porous network for GOx, higher conductivity	[61]
glucose	PQQ GDH ^6^-(p(MG)) NPs ^7^/GOx	0–2.5	10	5.5 ± 0.5	NR	NR	To orient the enzyme upon immobilization	[62]

^1^ LOD: limit of detection, ^2^ PEDOPT: PSS: poly (3,4-ethylenedioxythiophene) polystyrene sulfonate, ^3^ GOx: Glucose oxidase, ^4^ NR: not reported, ^5^ CMC-Na-GTAN: sodium carboxymethyl cellulose hydrogel-GOx-thioglycolic acid-gold nanoparticles, ^6^ PQQ GDH: pyrroloquinoline quinone dependent glucose dehydrogenase, ^7^ pMG NPs: poly (Methylene Green) nanoparticles.

**Table 4:** Diabetes wearable non-enzymatic electrochemical biosensors performance incorporating nanomaterials.

Detected Biomarker	Recognition Unit	Linear Concentration Range (mM)	LOD ^1^ (μM)	Sensitivity (μA mM^−1^ cm^−2^)	Reproducibility (%)	Recovery (%)	Nanomaterial Application	Ref.
glucose	CuNPs/PHEMA ^2^ hydrogel	0–0.2 & 0.2–4	9.99	2.5	3.4 (n = 5)	92	maximizes and maintains the active surface area for glucose interaction	[76]
glucose	nanoporous CuO ^3^, CuO/Ag, and CuO/Ag/NiO ^4^	0.001–5.50	0.1	2895.3	<2	96	the increased number of active sites and a larger surface area exposed to glucose and a rapid electron transfer and a low resistance to electron flow, leading to increased current density	[77]
glucose and lactate	PB-NPs ^5^ and nickel hexacyanoferrate nanozymes	0.001–2 for glucose	NR ^6^	(0.20 ± 0.01) × 10^6^	NR	NR	creating nanozymes-oxidase activity	[78]
glucose	Pt/MXene nanosheets	0–1	29.15	3.43	≤3	91.15	increases the active surface area and improve the current response to glucose	[79]
glucose	3D Ni-TMAF	0.1–2.5	0.33	203.89	0.73–4.9 (n = 3)	96.4–98.0	electrocatalytic activity towards glucose oxidation and efficient electron transfer and redox reactions	[80]
glucose	PU ^7^ fibrous mats and Pt nano-pine needles followed by magnetron sputtering of gold	0.1–4 and 5–10	14.77	203.13	NR	96.16–101.15	development of a stretchable, porous structure, with a large specific surface area	[81]
glucose	Cu_2_O/CuO NFs ^8^	higher than 2.5	0.0791	779	NR	NR	forming high surface area and selectivity, as well as the durability of mechanical deformation	[82]
glucose	CuO-LIG ^9^	0.08–1.5	80	2500	NR	92 ± 3	the large specific surface area with many groove structures is conducive to sweat transportation and storage	[83]
glucose	Cu/LIGF/LIG	0–4	0.124	1438.8	NR	NR	creating a larger surface area exposed to glucose, great stability	[84]
glucose	Fe_3_O_4_ nanospheres	0–18.0	19.2	96.1 ± 5.4	NR	≥95	provide favorable solid/liquid interface for mass diffusion and abundant active sites for sufficient oxidation of glucose	[85]
glucose	Polyaniline nanofiber	0.01–1	10.6	NR	NR	NR	scalable and cost-effective materials for electrode fabrication	[86]

^1^ LOD: limit of detection, ^2^ PHEMA: poly (2-hydroxyethyl methacrylate), ^3^ CuO: copper oxide, ^4^ NiO: Nickel oxide, ^5^ PB-NPs: Prussian blue nanoparticles, ^6^ NR: not reported, ^7^ PU: electrospun polyurethane, ^8^ Cu_2_O/CuO NFs: copper oxidase nanoflakes, ^9^ LIG: laser-induced graphene.

## Data Availability

Not applicable.

## Abbreviations

The following abbreviations are used in this manuscript:

CGM	continuous glucose monitoring
GOx	glucose oxidase
DET	direct electron transfer
LOD	Limit of detection
